# Supplementary material for: Changes in Meat of Hu Sheep during Postmortem Aging Based on ACQUITY UPLC I-Class Plus/VION IMS QTof
Source: Foods. 2024 Jan 4;13(1):174. doi: 10.3390/foods13010174 (PMC10778791; doi:10.3390/foods13010174)
Supplement: Supplementary file 1 [file foods-13-00174-s001.zip › foods-2726754-supplementary.pdf]

Table S1: Differential compounds identified in positive mode.

Compound	RT (min)	VIP	MIN P	MAX FC
7-Dehydrocholesterol	16.94	1.0845	1.54E-06	0.10403
Adenosine monophosphate	0.94	1.0692	5.07E-03	0.1461
Glycocholic acid	8.21	1.0486	3.81E-04	0.0922731
Hypoxanthine	1.60	1.2362	1.55E-10	2.6158
L-Proline	13.02	1.2902	6.60E-11	2.42194
Inosine	1.60	1.1783	1.13E-05	4.14642
Sphinganine	10.63	1.0807	1.87E-07	7.40301
3b,17b-Dihydroxyetiocholane	11.05	1.2203	3.33E-05	2.0505
3-Methylindole	8.06	1.2503	6.84E-05	0.437358
Indoleacrylic acid	3.50	1.1555	1.73E-06	2.33111
Undecanoic acid	10.19	1.4279	1.43E-10	3.56285
Enkephalin L	16.51	1.1233	1.98E-03	0.24478
N2-Succinyl-L-glutamic acid 5-semialdehyde	9.37	1.3988	3.65E-09	2.13017
N1-Acetylspermidine	14.68	1.2693	1.02E-03	3.82457
3-Methoxytyrosine	8.27	1.6412	4.88E-05	2.48698
Monoisobutyl phthalic acid	10.33	1.4437	3.13E-11	2.61366
Dicrocin	13.26	1.3181	4.68E-08	2.05789
Desmosterol	17.63	1.3727	1.88E-06	0.134854
LysoPC(18:1(9Z)/0:0)	9.80	1.1244	4.38E-07	3.44659
N-Methylnicotinamide	8.81	1.3320	1.85E-04	2.95787
Testosterone glucuronide	14.80	1.4248	5.75E-06	0.124385
Cinnamaldehyde	8.59	1.6872	2.64E-06	2.56298
Tryptophol	10.30	1.1046	2.51E-05	0.274331

(2E)-Tetradecenoyl-CoA	15.52	1.0385	1.37E-02	0.181034
Phytosphingosine	7.32	1.2752	1.55E-03	3.4605
9,10-DHOME	6.45	1.8056	3.35E-08	3.22173
FAPy-adenine	0.94	1.0645	4.08E-03	0.11126
Cer(d18:1/22:0)	17.65	1.2753	1.59E-04	0.000191585
Cer(d18:1/24:0)	15.27	1.1066	5.44E-06	0.00376505
Escitalopram	8.70	1.0356	1.66E-02	0.371297
TG(16:0/16:0/18:1(9Z))	17.62	1.3167	7.99E-13	0.138951
TG(16:0/18:0/18:2(9Z,12Z))	15.27	1.3851	1.30E-16	0.202441
Phenylacetaldehyde	8.58	1.6699	2.66E-07	5.34375
CPA(18:0/0:0)	9.68	1.3112	5.71E-08	6.5317
DG(14:1(9Z)/14:1(9Z)/0:0)	9.26	1.2055	1.01E-06	0.284772
DG(14:1(9Z)/16:1(9Z)/0:0)	9.69	1.3040	7.00E-09	0.0101556
LysoPA(0:0/18:0)	9.69	1.2091	5.74E-07	3.4318
PC(16:0/20:1(11Z))	13.03	1.3313	9.95E-08	0.0657534
PC(18:0/16:0)	13.04	1.4029	3.86E-06	0.244832
PC(18:2(9Z,12Z)/16:0)	11.76	1.2954	1.67E-04	0.0721756
PC(18:3(6Z,9Z,12Z)/22:6(4Z,7Z,10Z,13Z,16Z,19Z))	12.01	1.2578	7.08E-04	0.038114
PC(22:6(4Z,7Z,10Z,13Z,16Z,19Z)/20:4(5Z,8Z,11Z,14Z))	4.45	1.1355	1.54E-06	0.333592
PE(14:0/24:0)	4.90	1.2762	1.05E-08	0.391616
PE(14:1(9Z)/22:0)	12.22	1.1505	1.62E-06	5.60701
PE(15:0/22:0)	11.76	1.2211	5.31E-09	2.92555
PE(15:0/22:1(13Z))	11.76	1.2579	1.31E-08	3.48521
PI(16:0/18:0)	18.12	1.3937	1.07E-11	0.0399227
PI(16:0/20:2(11Z,14Z))	18.12	1.3449	1.72E-12	0.000608089
PI(16:0/22:4(10Z,13Z,16Z,19Z))	18.12	1.3288	1.47E-11	0.0166163

PI(18:0/22:4(10Z,13Z,16Z,19Z))	15.48	1.2545	1.99E-09	0.00179764
LysoPC(16:0/0:0)	9.52	1.2091	6.29E-09	3.27169
LysoPC(18:0/0:0)	10.85	1.0605	1.66E-04	2.02727
LysoPC(18:2(9Z,12Z)/0:0)	8.95	1.0389	1.01E-04	2.10668
LysoPC(20:0/0:0)	4.73	1.2112	9.47E-05	0.18359
LysoPC(P-16:0/0:0)	9.85	1.1504	3.33E-06	3.97018
LysoPE(0:0/18:0)	10.55	1.4784	2.70E-06	4.33194
LysoPA(O-18:0/0:0)	10.30	1.3642	6.94E-09	4.01606
LysoPE(P-16:0/0:0)	9.73	1.2188	1.91E-08	5.4446
gamma-Glutamylvaline	0.97	1.3523	2.33E-06	69.8801
PE(P-18:0/20:2(11Z,14Z))	11.76	1.2961	4.74E-10	0.0840917
PE(P-18:1(11Z)/20:3(8Z,11Z,14Z))	11.77	1.4050	1.00E-06	0.0841142
LysoPE(0:0/15:0)	9.90	1.1341	1.40E-07	151.176
MG(0:0/20:1(11Z)/0:0)	8.71	1.4157	2.17E-03	0.0520286
4-(Methylnitrosamino)-1-(3-pyridyl)-1-butanone	13.63	1.2845	3.09E-09	3.96652
Cer(d18:0/14:0)	11.49	1.5027	1.50E-08	2.21496
SM(d18:1/24:1(15Z))	12.62	1.4618	4.69E-13	0.0957293
Histidinal	13.30	1.3567	4.60E-13	3.15894
Methylarsonite	18.21	1.0455	6.45E-03	0.491093
10,11-Dihydro-12R-hydroxy-leukotriene E4	4.25	1.1935	4.23E-09	0.129024
Acetamidopropanal	13.11	1.2957	3.63E-11	2.23829
Neuromedin N (1-4)	10.33	1.0521	1.20E-02	0.0403297
Fumarycarnitine	12.03	1.4220	2.20E-12	3.65317
1b,3a,7b-Trihydroxy-5b-cholanoic acid	12.32	1.2471	6.70E-08	2.28968
Monoethylhexyl phthalic acid	10.32	1.3609	1.33E-11	2.44375
Pristanoylglycine	8.84	1.3402	1.43E-02	5.44038

Tryptophanamide	8.18	1.7118	2.61E-05	0.446641
3-Hydroxy-11Z-octadecenoylcarnitine	4.87	1.2456	1.57E-06	0.0318257
PC(O-22:2(13Z,16Z)/22:3(10Z,13Z,16Z))	13.15	1.0771	1.01E-03	0.121256
SM(d18:1/26:1(17Z))	16.34	1.2566	3.94E-07	0.0540868
PGP(18:1(11Z)/22:4(7Z,10Z,13Z,16Z))	11.01	1.1931	1.71E-08	0.000108293
4-Hydroxypropofol	11.77	1.1322	1.85E-06	2.1641
Azithromycin	11.96	1.3466	5.50E-07	5.44225
Ethambutol	12.90	1.3167	1.24E-12	3.11991
Eszopiclone	10.24	1.2702	3.65E-11	4.20995
Carteolol	9.69	1.0615	1.46E-08	5.10115
Docosanol	12.63	1.2690	1.09E-10	3.67974
Nystatin	12.40	1.1696	6.16E-10	0.0279784
Proparacaine	10.23	1.3057	1.84E-13	13.0974
Azlocillin	11.71	1.2460	5.78E-10	4.77165
Procarbazine	11.08	1.2308	3.10E-04	0.0283521
Fluoxymesterone	10.61	1.9808	2.31E-06	0.0242274
Arginylthreonine	17.22	1.3044	1.10E-06	4.26234
Glutaminylthreonine	4.25	1.2111	3.58E-07	0.0821329
Glycylvaline	11.91	1.1779	2.68E-08	129.546
Phenylalanylvaline	15.74	1.0491	1.33E-02	0.415649
Tryptophyl-Phenylalanine	11.76	1.7489	6.58E-06	0.487791
gamma2-Solamarine	14.98	1.2018	8.77E-04	0.187652
(2R,3R,4R)-2-Amino-4-hydroxy-3-methylpentanoic acid	10.54	1.2266	1.11E-08	2.3193
Torvoside C	4.78	1.0789	2.67E-04	0.206384
(4-Methylphenyl)acetaldehyde	8.60	1.6833	2.49E-07	2.2847
Cinnamyl alcohol	10.81	1.2869	2.36E-05	0.491555

L-Citronellol glucoside	11.30	1.3147	8.95E-12	2.3217
(3beta,22E)-26,27-Dinorergosta-5,22-dien-3-ol	15.61	1.0600	6.37E-05	0.390283
Artemidiol	7.50	1.0162	9.51E-06	0.131569
alpha,alpha-Dimethylanisalacetone	10.43	1.4143	7.45E-11	2.45622
5-Nonadecyl-1,3-benzenediol	12.64	1.5290	2.02E-06	2.66037
Annonisin	11.76	1.8678	1.25E-10	0.37835
Arachisprenol 11	16.18	1.9012	3.80E-07	0.00506078
Stearyl citrate	14.79	1.2723	1.17E-03	0.340148
1,1'-(Tetrahydro-6a-hydroxy-2,3a,5-trimethylfuro[2,3-d]-1,3-dioxole-2,5-diyl)bis-ethanone	9.00	1.2818	2.33E-07	0.0195493
Triethanolamine	13.39	1.3102	2.59E-12	2.33451
3-(2-Methylpropanoyloxy)-8-(2-methylbutanoyloxy)-9,10-epoxy-p-mentha-1,3,5-triene	15.10	1.6811	5.18E-05	0.069134
Lepidine B	8.81	1.2914	1.88E-07	0.299667
Osmaronin	12.67	1.3599	2.83E-13	4.31502
Methyl 15-cyanopentadecanoate	9.33	1.1661	2.39E-05	0.369112
alpha-Tocopherol succinate	4.61	1.1425	1.89E-05	0.306285
2-Diethylaminoethanol	18.12	1.3034	1.78E-12	2.08141
Withaperuvin H	5.56	1.9502	7.52E-07	4.48527
Apterin	6.83	1.2472	3.78E-07	0.459751
Digitoxigenin 3-[glucosyl-(1->6)-glucosyl-(1->4)-2,6-dideoxyribohexoside]	8.60	1.5232	4.61E-08	0.281788
Morellinol	10.68	1.4809	1.19E-03	4477.17
Methyl linoleate	13.02	1.2429	4.16E-12	2.22827
8,8-Diethoxy-2,6-dimethyl-2-octanol	10.43	1.4817	2.31E-11	3.69368
Tomatidine	10.01	2.0814	1.90E-07	2.11146
Ginsenoside Rc	5.01	1.3404	6.76E-12	0.31952
Ganoderic acid C1	11.88	1.3851	6.86E-07	2.36464

Squamone	15.56	1.4851	3.69E-04	0.241666
Ganoderic acid G	7.06	1.3979	2.79E-10	0.217817
Shyobunyl acetate	7.74	1.0098	5.05E-04	0.304581
2-(3-Phenylpropyl)pyridine	10.16	1.1067	7.78E-05	0.489613
Sclareol	18.12	1.2857	5.30E-10	0.00866076
7',8'-Dihydro-8'-hydroxycitraniaxanthin	10.81	1.3795	9.74E-07	0.212477
4-[(2-Methyl-3-furanyl)thio]-5-nonanone	12.04	1.6598	4.10E-04	12.9203
Butyl 2-decenoate	13.83	1.2720	2.78E-07	2.38113
Pentabromodiphenyl ethers	14.69	1.3333	1.30E-05	0.000156733
(E)-26,27-Dinorergosta-4,22-dien-3-one	10.52	1.0239	2.10E-03	3.34742
Petanin	10.99	1.2599	3.45E-07	0.105348
Isolimonic acid	11.89	1.3590	6.82E-04	2.21482
3-(Isothiocyanatomethyl)-1-methoxy-1H-indole	5.21	1.0242	3.39E-04	0.217863
Dilauryl 3,3'-thiodipropionate	4.97	1.3146	2.41E-12	0.1011
Polyporusterone C	12.60	1.1331	6.40E-06	2.33344
Glyuranolide	11.91	1.3467	5.15E-06	2.48445
Torvanol A	11.41	1.1534	2.10E-10	5.0051
Lupeoside	5.53	1.6884	2.78E-03	0.396134
Fenugreekine	0.92	1.0096	5.16E-07	0.0927939
Dihydrowyerone acid	8.59	1.6667	1.07E-06	2.83685
Dihydrowyerol	11.89	1.2975	2.21E-06	2.74358
13-Tetradecene-1,3-diyne-6,7-diol	10.07	1.1306	2.87E-07	2.6971
Cyclocalopin F	8.59	1.6785	4.79E-07	2.5045
(all-E)-6'-Apo-y-caroten-6'-al	13.05	1.0070	2.28E-03	0.124565
2,3-Dehydrosilybin	10.99	1.7215	1.46E-06	2.34593
Blumenol C glucoside	11.89	1.3302	1.63E-05	2.19385

(+)-Lyoniresinol 9-glucoside	8.60	1.6566	1.18E-07	5.71263
TG(16:0/16:0/22:4(7Z,10Z,13Z,16Z))	17.70	1.5048	1.15E-12	0.0212272
DG(18:2n6/0:0/18:2n6)	13.01	1.4858	3.08E-08	0.431062
CL(16:0/16:0/16:0/18:1(11Z))	5.09	1.2222	3.63E-05	0.344913
Casomorphin	8.59	1.3563	2.51E-03	0.390886
N-di-Demethyl roxithromycin	12.46	1.4646	1.37E-06	0.0302254
3-(3-Hydroxyphenyl)-2-methylpropionic acid	10.43	1.3833	8.09E-10	2.30014
Thiophene-4,5-epoxide	7.41	1.0184	2.42E-04	0.184928
4,5-Dihydro-drospirenone-3-sulfate	10.08	1.5168	6.79E-08	4.33228
Doxepin N-oxide glucuronide	13.49	1.0826	2.06E-06	2.52654
PC(DiMe(9,3)/DiMe(13,5))	14.72	1.0926	6.19E-04	0.123006
PS(MonoMe(11,5)/DiMe(11,3))	4.91	1.2434	1.50E-05	0.464931
3-hydroxypristanic acid	12.87	1.2564	1.19E-12	2.6073
Pentadecanoylcarnitine	7.54	1.0832	3.33E-03	0.0194697
N(1)-acetylsperminium(3+)	7.18	1.1773	5.57E-03	3.06723
N(1),N(12)-diacetylsperminium(2+)	17.07	1.4854	1.32E-08	0.386606
TG(20:0/i-12:0/i-18:0)	17.65	1.4049	1.05E-12	0.0633687
Leu-Leu-Leu	10.74	1.3700	1.26E-09	2.59503
PS(14:1(9Z)/20:5(5Z,8Z,11Z,14Z,17Z))	4.78	1.2628	3.55E-07	0.275991
PS(15:0/24:1(15Z))	11.01	1.0072	8.51E-06	0.00143887
PS(22:0/15:0)	11.43	1.2380	3.10E-05	0.310369
PE-NMe(18:2(9Z,12Z)/18:0)	12.01	1.3854	8.33E-06	0.11203
PE-NMe(18:3(6Z,9Z,12Z)/22:0)	11.01	1.2039	7.53E-04	1.93E-05
PE-NMe2(20:0/18:3(6Z,9Z,12Z))	4.92	1.0661	9.77E-05	0.455242
PE-NMe2(22:6(4Z,7Z,10Z,13Z,16Z,19Z)/22:6(4Z,7Z,10Z,13Z,16Z,19Z))	10.85	1.2309	4.72E-13	0.0281825
PA(22:2(13Z,16Z)/15:0)	18.11	1.1531	4.99E-05	0.118156

PA(22:4(7Z,10Z,13Z,16Z)/15:0)	4.48	1.2187	2.04E-08	0.129405
PA(24:0/22:2(13Z,16Z))	12.57	1.8311	7.17E-07	0.201049
PG(a-13:0/i-16:0)	14.02	1.2562	2.92E-03	2.26791
CL(8:0/25:0/a-25:0/a-25:0)	11.58	1.0535	5.29E-04	9.95E-05
CL(10:0/i-12:0/18:2(9Z,11Z)/18:2(9Z,11Z))	4.71	1.2692	1.06E-08	0.266301
CL(16:0/18:2(9Z,11Z)/18:2(9Z,11Z)/i-24:0)	11.76	1.3845	3.67E-05	0.146838
LysoPE(P-18:0/0:0)	10.95	1.2157	2.34E-08	5.49731
LysoPG(16:0/0:0)	11.93	1.0006	3.88E-08	6.65599
3-Hydroxyeicosanoylcarnitine	8.30	1.1400	1.39E-05	0.0693049
(4E,6Z)-3-Hydroxydeca-4,6-dienoylcarnitine	11.72	2.2794	3.11E-08	3.40201
3,8-Dihydroxydecanoylcarnitine	10.37	1.2633	6.00E-08	3.33821
(4Z)-3-Hydroxytetradec-4-enedioylcarnitine	10.92	1.3448	2.87E-11	2.34358
(9Z)-Nonadec-9-enoylcarnitine	14.69	1.3957	1.97E-15	0.00229722
2-Hydroxydocosanoylcarnitine	8.80	1.1274	8.43E-06	0.032374
Heptacosanoylcarnitine	14.41	1.0062	5.22E-03	0.107289
6-Hydroxynon-7-enoylcarnitine	12.12	1.1595	5.14E-04	0.296376
(5Z)-7-[(1R)-2-[(1E,3S)-3-Hydroxyoct-1-en-1-yl]-5-oxocyclopent-2-en-1-yl]hept-5-enoylcarnitine	8.88	1.4022	3.97E-06	0.185798
(9E,12Z)-10-Nitrooctadeca-9,12-dienoylcarnitine	9.97	1.0872	2.91E-06	4.08617
N-Palmitoyl Lysine	15.78	1.1486	7.21E-07	0.074059
N-Arachidonoyl Tryptophan	11.72	1.6795	8.27E-05	10.3138
N-Docosahexaenoyl Tryptophan	4.44	1.1756	1.42E-06	0.105722
N-Nervonoyl Proline	11.42	1.1038	1.61E-05	3.83734
(2S,3S,5S,8R,9S,10S,13S,14S,16S,17R)-17-Acetyloxy-10,13-dimethyl-2-morpholin-4-yl-16-(1-prop-2-enylpyrrolidin-1-ium-1-yl)-2,3,4,5,6,7,8,9,11,12,14,15,16,17-tetradecahydro-1H-cyclopenta[a]phenanthren-3-olate	4.95	1.1947	1.48E-08	0.13892

(-)-Morphinan	4.90	1.0276	1.88E-02	0.470222
((S)-2-((S)-2-((S)-2-((S)-1-(L-Prolyl-L-histidyl)pyrrolidine-2-carboxamido)-3-phenylpropanamido)-3-(1H-imidazol-4-yl)propanamido)-4-methylpentyl)-L-valyl-L-isoleucyl-L-histidyl-L-lysine	8.60	1.2255	9.89E-04	0.0417892
(1E)-1-Phenyltriaz-1-ene	8.58	1.6944	1.00E-08	9.01469
(1R,4R,4'R,5S,5'S,6R,6'S,7R,9R,11R,12R,13S,14S)-12-[(3R,4S,6R)-4-(Dimethylamino)-3-hydroxy-6-methyloxan-2-yl]oxy-4-ethyl-5,5',6,11-tetrahydroxy-4'-methoxy-4',5,6',7,9,11,13-heptamethylspiro[3,15,17-trioxabicyclo[12.4.0]octadecane-16,2'-oxane]-2,8-dione	12.20	1.8928	4.41E-06	2.2542
(1R)-3,5,5-Trimethyl-4-[3,7,12,16-tetramethyl-18-(2,6,6-trimethylcyclohexen-1-yl)octadeca-1,3,5,7,9,11,13,15,17-nonaenyl]cyclohex-3-en-1-ol	9.26	1.2146	1.45E-07	0.10675
(2-(4-((2-Chloro-4,4-difluoro-spiro(5H-thieno(2,3-C)pyran-7,4'-piperidine)-1'-yl)methyl)-3-methyl-pyrazol-1-yl)-3-pyridyl)methanol	0.74	1.0328	5.43E-04	0.202452
(2-Acetyloxy-3-hydroxypropyl) (E)-octadec-9-enoate	13.06	1.3798	7.24E-07	0.162503
Resiniferatoxin	13.26	1.0417	1.11E-07	2.28031
1,25-Dihydroxy-16-ene-vitamin D3	11.51	1.2081	1.95E-03	0.0739225
1-(6-((3-Methoxyestra-1,3,5(10)-trien-17-yl)amino)hexyl)-1H-pyrrole-2,5-dione	12.05	1.5535	1.93E-03	0.144069
2,4-Decadienamide,5-(4-methoxyphenyl)-N-[(1R)-1-methyl-4-(3-pyridinyl)butyl]-,(2E,4E)-	11.60	1.4462	4.78E-09	2.70976
13-Azaprostanoic acid	8.81	1.2958	1.48E-03	5.29695
N-(3-Aminopropyl)-N-methylcarbamic acid tert-butyl ester	12.90	1.2900	2.29E-11	2.5459
2-(1,2,3,4-Tetrahydroxybutyl)thiazolidine-4-carboxylic acid	10.32	1.1040	1.10E-04	0.366314
2-Aminobicyclo[2.2.1]heptane-2-carboxylic acid	2.77	1.0997	2.77E-04	2.59067
Mono(1-ethylhexyl) phthalate	10.32	1.4380	4.62E-10	3.31902
(5Z,7E)-9,10-Seco-5,7,10(19)-cholestatriene	17.64	1.3831	3.14E-07	0.261673
Tetraethylene glycol monododecyl ether	16.42	1.2478	3.21E-06	2.11011

6-n-Octylaminouracil	11.03	1.1684	2.85E-03	7.65E-09
Hexa-1,3,5-trienylbenzene	8.47	1.0271	1.63E-05	0.357242
N'-[(E)-[5-(Hydroxymethyl)-2-methyl-3-oxopyridin-4-ylidene]methyl]pyridine-4-carbohydrazide	5.54	1.6647	6.73E-06	4.44764
Albendazole-2-aminosulfone	12.45	1.2088	1.71E-09	15.0638
9-Octadecenamide, N-(2-hydroxyethyl)-, (9Z)-	16.36	1.1346	3.96E-07	0.109227
Blood group A antigen type 2	8.66	1.3414	9.25E-14	0.160711
(E)-4,4'-(Hex-3-ene-3,4-diyl)bis(4,1-phenylene) bis(dihydrogen phosphate)	18.08	1.0231	1.26E-06	5.64768
N-[1-[[5-(Diaminomethylideneamino)-1-oxopentan-2-yl]amino]-4-methyl-1-oxopentan-2-yl]-4-methyl-2-(propanoylamino)pentanamide	12.29	1.4897	2.37E-06	0.145636
s-Triazine, 2-amino-4-((4-methyl-1-piperazinyl)methyl)-6-piperidino-	9.96	1.1826	9.07E-08	4.44247
Alloferon	8.60	1.5423	1.00E-04	4.86209
Anisperimus	11.00	1.2354	9.11E-08	7.04036
Arabinofuranosylcytosine triphosphate	0.99	1.2112	4.35E-10	0.249474
Auristatin E	11.04	1.4557	1.16E-08	6.90722
[5-[2,4-Bis((3S)-3-methylmorpholin-4-yl)pyrido[2,3-d]pyrimidin-7-yl]-2-methoxyphenyl]methanol	14.75	1.1077	2.27E-05	2.93731
Benzodioxaphosphorin	8.31	1.0475	7.75E-04	0.342083
beta-Casomorphin-7	15.56	1.5022	4.06E-08	0.421324
Bimosiamose	8.59	1.6929	3.42E-05	2.65264
N,N,N',N'-Tetracyclohexyl-3-oxapentanediamide	12.62	1.0588	1.13E-07	3.53667
Cediranib	4.12	1.1194	1.30E-06	0.196896
Cetylmannoside	11.47	1.2842	9.27E-11	2.80941
1-O-Myristoyl-2-acetyl-glycerol	11.55	1.1642	6.16E-04	3.07378
3beta-Hydroxy-17-(1H-imidazol-1-yl)androsta-5,16-diene	12.18	1.2741	4.67E-07	0.011575
Ser-Leu-Ile-Gly-Lys-Val	4.42	1.2130	7.03E-08	0.379344

7-Nitro-2,3-Dioxo-2,3-Dihydroquinoxaline-6-Carbonitrile	8.31	1.0184	7.12E-04	0.106691
Chlorfenethol	14.40	1.0580	3.37E-04	2.18462
Chlorotrifluoroethylene	6.66	1.3262	8.51E-13	0.0110335
2-[[(1R)-2-[Bis(carboxymethyl)amino]cyclohexyl]-[(2S)-2-[bis(carboxymethyl)amino]-3-(4-isothiocyanatophenyl)propyl]amino]acetic acid	8.59	1.7283	1.69E-06	3.13058
Cicaprost	8.80	1.3476	3.25E-04	2.85012
Sulfamide, N,N-dimethyl-N'-((8alpha)-6-propylergolin-8-yl)-	4.12	1.1667	3.81E-07	0.124279
Cyclo(Arg-Gly-Asp-D-Phe-Val)	7.67	1.1199	4.57E-05	0.35821
Decachlorobiphenyl	5.50	1.5582	4.88E-06	2.17734
Delapril	8.59	1.6784	6.13E-07	2.37897
Desidustat	10.07	1.5486	8.35E-10	2.34088
1,1-Bis(2-aminoethyl)-2-hydroxy-3-oxotriazane	13.28	1.2321	5.31E-10	7.94227
Dibekacin	18.12	1.3934	6.09E-11	0.00235107
Diethylaminoethyl-Sephacel	13.35	1.2637	1.24E-11	3.74757
Doqualast	8.60	1.7083	9.90E-07	3.65417
Dropropizine	11.91	1.2461	1.56E-02	2.01328
Enalkiren	11.76	2.2691	1.12E-09	3.01865
ethyl ((3,4,5-trihydroxy-6-((4-(4-isopropoxybenzyl)-1-isopropyl-5-methyl-1H-pyrazol-3-yl)oxy)tetrahydro-2H-pyran-2-yl)methyl) carbonate	10.88	1.4066	7.52E-05	5.96324
Phenethyl	8.59	1.6644	3.17E-08	2.14921
Exametazime	10.15	1.3066	2.19E-12	5.49924
Floctafenic acid	17.68	1.2175	5.66E-11	6.3033
Fukugiside	8.89	1.2175	7.20E-07	0.0388741
Glucose pyruvate	8.59	1.7225	1.17E-07	3.16766
3-(3-(N-(2-Chloro-3-trifluoromethylbenzyl)(2,2-diphenylethyl)amino)propoxy)phenylacetic acid	12.88	1.0517	2.98E-08	4.22607

Val-Gly-Val-Ala-Pro-Gly	12.62	1.1154	4.72E-07	4.47198
Hetastarch	0.74	1.1172	4.69E-06	0.124073
N,N-Diallyl-tyrosyl-aminoisobutyryl-aminoisobutyryl-phenylalanyl-leucine	13.64	1.1077	8.31E-06	0.4055
l-lysyl-l-lysyl-l-lysine	14.15	1.0857	3.65E-05	2.78967
Leptine I	13.68	1.2075	1.19E-05	0.00196321
Lestaurtinib	12.00	1.2168	8.51E-09	3.88899
linoleoyl-dioleoyl-glycerol	18.10	1.2989	1.18E-06	0.0516264
Lurasidone	18.01	1.3209	1.31E-03	0.188273
(3-{[3-(2-Amino-2-Oxoethyl)-1-Benzyl-2-Ethyl-1h-Indol-5-Yl]oxy}propyl)phosphonic Acid	8.59	1.6883	2.52E-05	2.06347
1-Methyl-5-(4-benzoyl)pyrrole-2-acetic acid 2-(theophylline-7-yl)ethyl ester	8.09	1.1789	4.36E-03	2.79254
Methyl 20-dihydroprednisolonate	10.68	1.5381	5.04E-04	335.918
Milbemycin A3	10.68	1.5139	4.83E-04	301.663
Milbemycin beta1	12.82	1.5242	3.95E-03	0.199917
Midecamycin acetate	12.50	1.3226	4.05E-06	0.140321
Avemectin B1	4.90	1.2206	4.17E-06	0.49619
Dimercaptosuccinic acid monomethyl ester	7.23	1.4709	1.51E-08	2.83455
Mycobactin J	10.95	1.2777	3.78E-06	0.098187
N-Acetyl-S-benzyl-L-cysteine	8.63	1.0738	9.65E-05	0.0620085
N-Desethyl Sunitinib	9.72	1.1329	4.14E-03	0.000231722
N-Methacryloyl-L-glutamic acid	8.71	1.8808	5.71E-04	2.29707
N-Methoxysuccinyl-Ala-Ala-Pro-Val	9.00	1.1029	2.56E-03	0.0235405
N(4)-Oleylcytosine arabinoside	10.29	1.1348	4.87E-06	5.61136
(2R)-1-(4-Nonylphenyl)propan-2-amine	13.56	1.2925	4.94E-08	0.27505
1,1,1-Trifluorohenicosa-6,9,12,15-tetraene	4.78	1.2462	9.02E-07	0.216131
NeuAc(alpha->6)GalNAc(alpha1->O)Ser	9.75	1.2653	1.04E-08	0.419323

oleandomycin	11.55	2.2712	1.82E-07	4.94682
(2S)-2-[[(2R)-3-[2,3-Di(hexadecanoyloxy)propylsulfanyl]-2-(hexadecanoylamino)propanoyl]amino]-3-hydroxypropanoic acid	4.71	1.1399	3.59E-04	0.328213
1,1,1-Trifluoroheptadecan-2-one	10.26	1.1129	6.22E-08	19.4292
3-(4-Iodophenyl)-2-mercapto-(Z)-2-propenoic acid	18.15	1.2897	9.16E-09	2.00846
Pentisomide	13.97	1.3063	2.43E-06	0.13729
Phenylphosphonic acid	9.90	1.1699	2.33E-05	0.316912
Phosphoramidon	11.41	1.0575	8.80E-09	9.05473
Phytantriol	9.37	1.2849	6.66E-04	7.85831
Reproterol	6.99	1.5173	7.14E-05	123.473
1-(2-(1-Adamantyl)ethyl)-1-pentyl-3-(3-(4-pyridyl)propyl)urea	11.72	1.7552	2.26E-08	0.130962
Sar chelate	11.72	1.2791	3.40E-11	6.54541
3,3-Bis(carboxymethyl)hexadecanedioic acid	10.92	1.4003	4.58E-12	2.75991
9-(Formyloxymethyl)-9H-fluorene-2-sulfonic acid	9.18	1.1716	8.69E-07	2.81443
Methylglyoxal-bis-guanylhydrazone	11.31	1.4110	2.19E-09	2.92173
(1R,2S,4S,6R,7S,8R,9S,12S,13S)-7,9,13-Trimethyl-6-(3-methylbutyl)-5-oxapentacyclo[10.8.0.02,9.04,8.013,18]icosan-16-ol	15.66	1.2617	3.52E-08	0.358134
Solasonine	13.64	1.2972	1.60E-10	0.106242
3-((((-Oxoheptyl)amino)acetyl)amino)methyl-7-oxobicyclo(2.2.1)hept-2-yl-5-heptenoic acid	10.11	1.0479	3.83E-08	5.14413
Streptidine	8.47	1.0420	2.64E-05	0.438166
Tetranorprostanedioic acid	12.31	1.3350	1.79E-13	2.76352
TETRATHIONIC ACID	18.17	1.1311	1.21E-03	0.150101
Candidin	11.01	1.2066	1.36E-04	0.0405775
N,N-Diisopropyl-N'-isoamyl-N'-diethylaminoethylurea	11.68	1.3196	8.83E-11	2.23997
VAPIPROST	14.13	1.4307	1.80E-12	0.00903729

ZORUBICIN	16.93	1.0252	2.06E-04	3.99213
4-((4-(2-(6-((2R)-2-(2,4-Difluorophenyl)-1,1-difluoro-2-hydroxy-3-(1H-tetrazol-1-yl)propyl)-3-pyridinyl)ethynyl)phenoxy)methyl)benzonitrile	5.58	1.1121	3.01E-09	4.20112
PE(LTE4/18:0)	13.69	1.6820	5.78E-06	0.00903752
PE(20:0/PGF1alpha)	11.90	1.1822	1.04E-05	0.437786
PA(20:0/LTE4)	11.66	1.2776	8.51E-05	0.000637439
PA(20:1(11Z)/LTE4)	13.40	1.1779	2.26E-08	5.87E-05
PA(20:2(11Z,14Z)/LTE4)	13.18	1.0772	1.18E-03	0.49175
PA(LTE4/20:2(11Z,14Z))	12.03	1.4280	4.24E-10	0.175886
PA(LTE4/22:1(13Z))	15.10	1.1767	4.66E-05	0.0026517
PA(22:6(4Z,7Z,10Z,13Z,16Z,19Z)/PGE2)	11.69	1.3384	1.74E-05	0.0420775
PA(PGE2/22:6(4Z,7Z,10Z,13Z,16Z,19Z))	12.73	1.3092	3.39E-05	0.143043
PA(a-25:0/20:4(7E,9E,11Z,13E)-3OH(5S,6R,15S))	10.30	1.9110	6.41E-07	0.136032
PG(a-17:0/PGF1alpha)	13.64	1.6185	7.40E-08	0.163744
PG(a-21:0/PGF2alpha)	15.69	1.4452	1.39E-08	0.038785
PGP(20:3(8Z,11Z,14Z)-O(5,6)/a-13:0)	4.78	1.2500	3.65E-07	0.421202
PGP(i-14:0/20:3(8Z,11Z,14Z)-2OH(5,6))	11.05	1.3674	6.16E-06	0.00252928
PI(PGF1alpha/16:0)	10.82	1.1136	5.36E-05	0.162605
PI(18:0/20:4(6E,8Z,11Z,14Z)-OH(5S))	11.69	1.4488	3.85E-07	0.00162038
PI(20:0/18:1(12Z)-2OH(9,10))	13.69	1.0887	9.84E-07	0.11204
PI(20:0/20:3(6,8,11)-OH(5))	14.64	1.4335	1.67E-09	0.160579
PS(18:1(12Z)-2OH(9,10)/18:0)	13.86	1.2524	3.41E-05	0.139842
PS(20:5(7Z,9Z,11E,13E,17Z)-3OH(5,6,15)/18:3(6Z,9Z,12Z))	13.42	1.2903	4.42E-09	0.11868
PS(PGJ2/20:1(11Z))	13.69	1.3364	6.55E-06	0.0528001
PS(22:0/20:4(6Z,8E,10E,14Z)-2OH(5S,12R))	15.49	1.2763	1.63E-11	0.00395003
PS(20:3(8Z,11Z,14Z)-2OH(5,6)/22:0)	15.56	1.2902	3.90E-13	0.308677

PS(22:1(13Z)/PGF1alpha)	15.10	1.2222	8.15E-06	0.0570995
PS(24:0/PGF2alpha)	14.63	1.1891	1.39E-03	0.0134708
PS(PGF2alpha/24:0)	15.10	1.1328	2.37E-03	0.0256448
PS(20:3(8Z,11Z,14Z)-2OH(5,6)/24:0)	15.55	1.0138	6.14E-03	0.0227572
PS(24:0/5-iso PGF2VI)	14.12	1.2894	6.97E-11	0.00513811
PS(5-iso PGF2VI/24:0)	14.64	1.3075	4.44E-11	0.166674
PE(22:0/PGJ2)	11.39	1.0288	2.30E-06	0.00200735
PE(22:2(13Z,16Z)/22:5(4Z,7Z,10Z,13Z,19Z)-O(16,17))	4.91	1.3553	4.81E-12	0.00826302
PE(24:0/20:3(6,8,11)-OH(5))	5.00	1.2448	1.06E-04	0.0217414
PE(LTE4/P-16:0)	18.12	1.4061	5.43E-13	0.00390561
PC(15:0/18:2(10E,12Z)+=O(9))	18.11	1.3590	2.46E-05	0.0335241
PC(16:0/TXB2)	4.45	1.0591	4.53E-06	0.247436
PC(20:3(5Z,8Z,11Z)/6 keto-PGF1alpha)	14.76	1.1761	1.95E-04	0.00993139
PC(22:6(4Z,7Z,10Z,13Z,16Z,19Z)/PGJ2)	18.10	1.0311	1.16E-05	0.0485146
PC(22:6(4Z,7Z,10Z,13Z,16Z,19Z)/20:4(6E,8Z,11Z,14Z)+=O(5))	18.17	1.2186	2.18E-05	0.458302
PC(24:0/PGF2alpha)	4.31	1.1135	1.60E-04	0.0608814
PC(DiMe(9,3)/5-iso PGF2VI)	12.03	1.3020	3.14E-05	0.078644
PC(P-18:0/20:4(6E,8Z,11Z,14Z)-OH(5S))	13.04	1.2926	9.04E-05	0.0558849
PC(P-18:0/PGF2alpha)	4.76	1.3667	1.83E-09	0.00748857
PC(20:3(6,8,11)-OH(5)/P-18:0)	12.01	1.1688	8.92E-04	0.000120354
Cer(d16:1/PGJ2)	10.99	1.0825	2.43E-02	2.30291
Cer(d20:1/18:1(12Z)-2OH(9,10))	13.88	1.2528	2.66E-04	6.58269
Cer(t18:0/5-iso PGF2VI)	18.10	1.3070	8.29E-08	0.0151203
SM(d17:1/20:3(5Z,8Z,11Z)-O(14R,15S))	5.58	1.3443	4.68E-05	0.0107848
SM(d17:1/20:5(7Z,9Z,11E,13E,17Z)-3OH(5,6,15))	10.58	1.0506	2.28E-04	4.53E-05
SM(d18:0/20:3(6,8,11)-OH(5))	5.57	1.3550	1.02E-08	2.54978

SM(d19:0/LTE4)	5.01	1.2844	8.42E-08	0.185906
SM(d20:1/20:4(5Z,8Z,11Z,14Z)-OH(19S))	13.04	1.2628	8.75E-08	0.111535
SM(d20:1/LTE4)	12.01	1.7221	6.79E-06	0.0173698
DG(10:0/20:5(7Z,9Z,11E,13E,17Z)-3OH(5,6,15)/0:0)	10.28	2.0080	5.97E-05	0.165734
DG(12:0/6 keto-PGF1alpha/0:0)	10.91	1.3769	1.31E-07	0.247097
DG(13:0/20:4(6Z,8E,10E,14Z)-2OH(5S,12R)/0:0)	15.34	1.3455	8.43E-04	0.147771
DG(15:0/20:3(8Z,11Z,14Z)-2OH(5,6)/0:0)	8.66	1.2083	2.19E-05	0.224315
DG(20:4(6E,8Z,11Z,13E)-2OH(5S,15S)/15:0/0:0)	16.43	1.3309	1.66E-04	0.254459
DG(22:6(4Z,7Z,11E,13Z,15E,19Z)-2OH(10S,17)/15:0/0:0)	13.88	1.2327	5.11E-05	2.65255
DG(17:0/6 keto-PGF1alpha/0:0)	4.32	1.2301	7.67E-08	0.396765
DG(17:0/PGF1alpha/0:0)	4.80	1.3202	4.97E-12	0.449314
DG(18:0/20:3(6,8,11)-OH(5)/0:0)	18.12	1.3177	3.67E-14	0.0441212
DG(8:0/20:3(8Z,11Z,14Z)-2OH(5,6)/0:0)	15.56	1.5820	3.57E-06	0.433686
DG(PGF2alpha/a-25:0/0:0)	10.30	1.0117	1.95E-04	0.0320619
DG(a-25:0/PGJ2/0:0)	11.76	1.3587	2.74E-08	0.122014
DG(a-25:0/PGF1alpha/0:0)	12.21	1.5048	1.83E-08	0.00227502
DG(PGF1alpha/a-25:0/0:0)	13.35	1.4432	3.95E-07	0.000858881
Serylvalylglycylglutamic acid	0.75	1.1530	1.05E-03	25.2493
Psilostachyin	8.59	1.6650	4.02E-07	3.72582
2,4-Dimethylquinoline	9.93	1.3121	4.96E-05	0.032204
alpha-Allocryptopine	9.90	1.0773	4.83E-09	7.99434
Celosianin	10.27	1.4776	7.40E-04	2.31927
Lactupicrin methyl ester	8.59	1.3453	1.16E-03	0.149436
Aluminum oleate	18.09	1.3557	2.59E-07	0.0133702
Calcium oleate	10.77	1.1652	4.59E-07	0.398378
Poly(acrylic acid-co-hypophosphite), sodium salt	6.57	1.2511	7.27E-09	0.0215291

Polyglycerol esters of fatty acids	13.05	1.2953	1.02E-11	3.17129
Sodium palmitate	10.15	1.2995	2.83E-10	2.67924
Sodium stearyl fumarate	9.42	1.0141	4.52E-07	6.22039
Succistearin	5.40	2.0190	5.86E-05	3.81891
Magnesium dodecanoate	12.62	1.0453	8.99E-07	4.59812
Curvacin A	4.82	1.3257	1.27E-12	0.0492153
[Ethylenebis(dithiocarbamato)]manganese	18.18	1.3131	4.54E-08	0.136216
8'-Apo-b-caroten-8'-ol	4.92	1.3188	1.96E-11	0.317257
2-Hydroxyglutaric acid diethyl ester	12.31	1.3065	1.23E-11	2.15427
octanoate	7.63	1.2354	3.20E-09	0.293787
Cyanidin 3-O-(6''-dioxalyl-glucoside)	17.62	1.0637	8.62E-04	3.68E-05
Asn-Arg-Ala-Ile	17.21	1.2881	5.24E-09	5.73982
Lys-Asp-Tyr	6.62	1.4644	4.26E-05	0.137975
Gum arabic	9.24	1.2287	9.46E-04	0
Serdexmethylphenidate	5.50	1.6923	4.80E-06	2.03923
Avapritinib	11.89	1.3406	6.51E-06	2.0299
n-decanohydroxamic acid	12.63	1.3181	4.76E-10	2.63773
Valproic acid	10.48	1.4104	3.14E-09	2.27628
4,7,10,13,16,19-Docosahexaynoic acid	9.68	1.0248	9.44E-04	2.3738
5,8,11,14-Octadecatetraynoic acid	8.60	1.6751	5.26E-07	2.75361
4-Methyl-3Z,5-hexadienoic acid	8.59	1.6997	1.45E-08	3.55058
26:6(8Z,11Z,14Z,17Z,20Z,23Z)	10.54	1.0205	9.41E-03	0.489934
methyl 8-[3,5-epidioxy-2-(3-hydroperoxy-1-pentenyl)-cyclopentyl]-octanoate	8.18	1.3751	1.38E-06	0.217291
6-bromo-23-methyl-tetracosa-5E,9Z-dienoic acid	15.46	1.0686	1.61E-04	2.40289
Oncobic acid	9.52	1.0841	1.51E-06	7.68757
alpha,alpha'-Trehalose 6-mycolate	11.73	1.0273	8.41E-04	0.0432153

Tetranor-PGF1alpha	11.76	1.7197	7.58E-09	0.313842
Linoleyl palmitate	10.62	1.2498	6.89E-10	0.269559
3-acetyl-3-methyldihydrofuran-2(3H)-one	11.89	1.2986	3.42E-05	2.1739
N,N-(2,2-dihydroxy-ethyl) arachidonoyl amine	12.63	1.2927	1.99E-11	3.50675
(+/-)N-(2-fluro-ethyl)-2,16,16-trimethyl-5Z,8Z,11Z,14Z-docosatetraenoyl amine	16.85	1.1832	4.17E-07	0.499082
N-oleoyl tryptophan	12.03	1.6427	7.76E-04	20.8673
Anandamide (20:2, n-6)	10.52	1.3649	2.73E-10	2.20396
Anandamide (20:l, n-9)	12.47	1.3461	8.34E-12	3.59931
Oleoyl-EA(d2)	12.82	1.1351	3.40E-07	2.26548
9Z,19Z-Heptatriacontadiene	7.06	1.0945	3.56E-04	0.174874
1-O-alpha-D-glucopyranosyl-(2-tetradecanoyloxy)-eicosan-1-ol	14.81	1.1166	1.74E-04	0.324963
DG(20:5(5Z,8Z,11Z,14Z,17Z)/0:0/20:5(5Z,8Z,11Z,14Z,17Z)) (d5)	14.33	1.1212	5.01E-05	0.189746
DG(20:4(5Z,8Z,11Z,14Z)/0:0/20:4(5Z,8Z,11Z,14Z)) (d5)	18.12	1.2702	1.54E-07	0.160799
DG(18:0/0:0/18:0) (d5)	11.05	1.1027	3.10E-05	0.0202191
DG(O-16:0/18:1(9Z))	13.66	1.1011	2.27E-03	2.11238
1-(14-methyl-pentadecanoyl)-2-(8-[3]-ladderane-octanyl)-sn-glycerol	18.12	1.3360	2.17E-15	0.0319352
TG(20:2(11Z,14Z)/18:3(6Z,9Z,12Z)/20:2(11Z,14Z)) (d5)	16.22	1.2794	1.79E-08	0.024853
TG(20:4(5Z,8Z,11Z,14Z)/18:2(9Z,12Z)/20:4(5Z,8Z,11Z,14Z)) (d5)	16.15	1.0967	8.19E-11	0.0321281
SQDG(22:5(5Z,8Z,11Z,14Z,17Z)/16:1(13Z))	4.90	1.1762	2.92E-05	0.35794
PC(18:1(17Z)/18:1(17Z))	11.77	1.2023	3.68E-08	0.0612193
PC(22:0/22:1(13Z))	12.14	1.1250	1.32E-03	0.305774
PC(O-14:0/18:0)	11.56	1.4135	4.04E-11	8.74469
PC(O-6:0/O-6:0)	11.52	1.0081	1.82E-05	2.3187
1-(6-[3]-ladderane-hexanoyl)-2-(8-[3]-ladderane-octanyl)-sn-glycerophosphocholine	16.42	1.2073	1.13E-05	0.448109
PE(21:0/22:6(4Z,7Z,10Z,13Z,16Z,19Z))	13.06	1.1928	6.81E-04	0.00621421
PE(18:4(6Z,9Z,12Z,15Z)/18:4(6Z,9Z,12Z,15Z))	4.64	1.2228	3.99E-08	0.203704

PE(19:0/18:1(9Z))	11.76	1.4000	5.39E-06	0.184222
PE(P-19:1(12Z)/0:0)	12.36	1.1385	8.04E-03	0.0742887
PS(19:0/20:4(5Z,8Z,11Z,14Z))	10.26	1.3205	1.45E-04	0.000984289
PG(14:1(9Z)/14:1(9Z))	15.34	1.3103	7.40E-04	0.456334
PG(O-18:0/14:0)	12.22	1.7869	2.44E-08	2.60268
PG(12:0/0:0)	9.00	1.6100	2.61E-06	3.76354
PG(22:4(7Z,10Z,13Z,16Z)/0:0)	15.99	1.3735	1.04E-06	0.0325794
PI(18:2(9Z,12Z)/22:1(11Z))	15.56	1.2414	1.60E-07	0.297844
PI(19:1(9Z)/19:1(9Z))	14.70	1.2894	1.97E-06	0.108473
PI(16:0/20:3(8Z,11Z,14Z))	14.72	1.2396	5.62E-06	0.274548
PI(O-16:0/13:0)	10.18	1.0478	1.22E-02	0.0187472
PI(O-18:0/21:0)	11.26	1.4642	3.99E-06	2.09301
PI(O-20:0/22:0)	4.57	1.1067	3.61E-05	0.209348
Archaetidylglycerol-myo-inositol	5.00	1.2495	5.54E-07	0.245773
PA(17:0/22:6(4Z,7Z,10Z,13Z,16Z,19Z))	4.53	1.1307	6.82E-08	0.139656
PA(O-18:0/17:0)	11.76	1.2362	2.11E-07	0.284243
PA(O-18:0/18:3(6Z,9Z,12Z))	18.11	1.0797	7.82E-03	0.007093
PA(O-20:0/17:2(9Z,12Z))	13.04	1.3691	3.25E-09	4.14281
PA(O-16:0/20:5(5Z,8Z,11Z,14Z,17Z))	12.13	1.2736	4.01E-06	0.219152
N-(3E-hexadecenoyl)-deoxysphing-4-enine-1-sulfonate	10.81	1.2034	3.60E-07	0.420348
Prosopinine	17.65	1.4191	7.22E-06	0.280407
N,N-dimethyl-Safingol	12.01	1.3488	1.85E-10	4.56876
LacCer(d18:1/24:1(15Z))	18.12	1.0382	1.35E-02	0.00277453
GalCer(d18:1/24:0)	15.23	1.1803	2.26E-09	0.000196077
GalCer(d18:1/26:1(17Z))	18.10	1.2886	5.19E-08	0.0321327
(3'-sulfo)Galbeta-Cer(d18:1/16:0(2OH))	18.10	1.4476	6.06E-09	0.00503569

(3'-sulfo)Galbeta-Cer(d18:0/18:0(2OH))	18.17	1.1876	1.47E-06	0.00479343
C24:1 Sulfatide	13.19	1.2215	9.00E-06	0.31692
